# Feasibility Study of the Electromagnetic Damper for Cable Structures Using Real-Time Hybrid Simulation

**DOI:** 10.3390/s17112499

**Published:** 2017-10-31

**Authors:** Ho-Yeon Jung, In-Ho Kim, Hyung-Jo Jung

**Affiliations:** Korea Advanced Institute of Science and Technology, Department of Civil and Environmental Engineering, KAIST, 291 Daehak-ro, Yuseong-gu, Daejeon 34141, Korea; hyjung89@gmail.com (H.Y.J.); kih119@kaist.ac.kr (I.H.K.)

**Keywords:** electromagnetic (EM) damper, cable vibration control, energy harvesting, hybrid simulation

## Abstract

Cable structure is a major component of long-span bridges, such as cable-stayed and suspension bridges, and it transfers the main loads of bridges to the pylons. As these cable structures are exposed to continuous external loads, such as vehicle and wind loads, vibration control and continuous monitoring of the cable are required. In this study, an electromagnetic (EM) damper was designed and fabricated for vibration control and monitoring of the cable structure. EM dampers, also called regenerative dampers, consist of permanent magnets and coils. The electromagnetic force due to the relative motion between the coil and the permanent magnet can be used to control the vibration of the structure. The electrical energy can be used as a power source for the monitoring system. The effects of the design parameters of the damper were numerically analyzed and the damper was fabricated. The characteristics of the damper were analyzed with various external load changes. Finally, the vibration-control and energy-harvesting performances of the cable structure were evaluated through a hybrid simulation. The vibration-control and energy-harvesting performances for various loads were analyzed and the applicability to the cable structure of the EM damper was evaluated.

## 1. Introduction

In Korea, structural accidents, such as the collapses of the Gyeongju Maury Resort (2014) and the Busan North—South Bridge (2013), occur frequently. Recently, the cable fire accident of the Seohae Grand Bridge (2015) caused a casualty, and consumed considerable time and cost to recover the bridge. Annually, various structural failures cause casualties and financial losses. In order to prevent the structural failure, vibration control and monitoring systems are applied to various civil structures. The bridge is a representative civil engineering structure that not only plays a role in contributing to economic growth by utilizing land more efficiently, but also enables the balanced development of national industries and regions. With the continuous development of construction technology, the rate of the construction of cable bridges, such as cable-stayed and suspension bridges, is increasing. It is necessary to continuously maintain and monitor a bridge cable because the cable, which is a main component of the cable bridge, transfers various loads to the main tower by tension of the cable. Due to the characteristics of the cable structures, the bridge cables have a very low damping ratio. These low damping ratios cause fatigue loads on the cables, by continuous exposure to wind and vehicle loads. The fatigue loads acting on the cable lead to the deterioration of the lifetime of the bridge. Therefore, vibration control and monitoring of the cable are required. The electromagnetic (EM) damper, which has both vibration-control and energy-harvesting functions, has been proposed as an effective maintenance method for the cable structure. The EM damper can reduce the vibration of the cable to an acceptable level and obtain the power of the monitoring sensor through the energy-harvesting function. In addition, it can respond appropriately to typhoons and earthquakes.

Recently, research on the regenerative damping system, which is a method of controlling vibrations of a structure and utilizing the energy generated in a vibration-control process, has been actively conducted. A regenerative damping system converts vibration energy into electric energy, providing a process for the vibration control of a structure [1,2,3,4,5,6]. Because the extra energy obtained from the control process can be utilized for various purposes, studies have been conducted to apply a regenerative damping system to the civil engineering field [7,8,9].

Studies on vibration-control devices combined with electromagnetic devices have been performed. Nakamura (2014) proposed an electromagnetic (EM) damper, combined with a rotating inertial mass (EIMD). The EIMD is able to generate a large inertial force created by the rotating flywheel and a variable damping force produced by the electric generator. The EIMD is able to reduce story drifts as well as accelerations, and it surpasses conventional types of dampers in reducing acceleration responses [10]. Amjadian (2016) proposed a passive electromagnetic eddy current friction damper that combines an eddy current damper and a friction damper. A numerical analysis was conducted by combining the eddy current damper and friction. The vibration-control performance of the proposed damper corresponded to the existing magnetorheological (MR) damper [11].

Arias (2005) proposed a passive electromagnetic damping device for the vibration control for building structures [12]. Throughout the design process of the EM damper, a tubular permanent magnet linear machine was developed. In the design process of the EM damper, validation of the mathematical model of the damper was conducted with a small-scale prototype EM damper. The feasibility of the system was performed with a full-scale simulation building model. Arias et al. (2011) investigated the feasibility of the electromagnetic damping systems. The feasibility analysis was conducted to validate the physical and economic viability of the EM damper as a structure vibration-control device [13]. This device presented several advantages over other passive structural dampers, such as energy dissipation external to the device, possible operation as a semi-active damper by modifying the circuit impedance, and possible operation as an actuator by reversing the energy flow direction [13]. Shen and Zhu (2015) also studied energy-harvesting strategies using the EM damper in a bridge stay cable [14]. Using the EM damper and a buck-boost circuit, which is an energy-harvesting circuit, the energy-harvesting and vibration-control performance was evaluated with the numerical simulations.

Shen (2014) proposed an electromagnetic damping and energy-harvesting (EMDEH) device, consisting of an EM damper and energy-harvesting circuit [15]. In the study, a numerical model of an EM damper and energy-harvesting circuit was constructed. In the design process of the proposed device, the efficiency of the EM damper and energy-harvesting circuit was observed. Finally, the optimized EMDEH device was evaluated with numerical simulations of the tall building structure and bridge stay cable, considering wind excitation. From the numerical simulations, the proposed device implied that it is feasible for full-scale civil structures.

Recently, Shi (2017) proposed a passive negative stiffness damper based on the magnetic negative stiffness damper (MNSD) [16]. For the cable structure, the vibration-control performance of the adjustable magnetic negative stiffness damper was numerically and experimentally validated. By adjusting the negative stiffness, the cable vibration mitigation performance was evaluated. As the value of the negative stiffness increased, and by considering the random and harmonic loadings, the performance of the damper improved.

Research on the application of electromagnetic devices to cable structures has been actively conducted. However, most studies have only presented numerical analyses. Although the EM damper has been applied to laboratory scale reduction models, and the vibration-control performance has been evaluated, the EM damper behavior has not yet been analyzed. Moreover, only high-speed wind loads that generate excessive vibration of the structures have been considered, and various low-speed wind loads have been overlooked. In order to evaluate the applicability of the EM damper, it is necessary to consider the characteristics of the EM damper as well as the wind loads at various speeds.

This study aims to evaluate the feasibility of the EM damper for vibration-control and structure monitoring. In this study, a tubular-type linear damper was first fabricated as a vibration-control method of a cable structure, and characteristic and performance tests were conducted. The proposed EM damper has capabilities of vibration control and energy harvesting, which can be used as a source of the energy for the monitoring system. The characteristics of the damper were analyzed through characteristic tests. For the various external loads, the variation of the damper force was measured. Before applying the damper to the target structure, the behavior of the damper according to the external load was investigated. A real-time hybrid simulation was performed to evaluate the applicability of the damper for the cable structure. Contrary to previous studies, the performance of the EM damper was analyzed using a hybrid simulation incorporating the bridge cable properties to reflect the actual characteristics of the cable structure. The vibration-control and energy-harvesting performances of the cable structure were evaluated through a hybrid simulation that reflected the characteristics of the actual damper, rather than a numerical analysis reflecting the ideal damper model. In order to analyze the energy-harvesting performance of the EM damper, the mean wind speed acting on the actual bridge was considered.

## 2. Electromagnetic (EM) Damper Design

An EM damper is a typical regenerative damping system and has been widely used for automobile vibration control. The regenerative damping utilizes the electromagnetic force generated during the process of converting mechanical vibration energy of a structure into usable electrical energy as system damping. The regenerative damping is used in various fields as the utilization of regenerative energy generated in the vibration-control process increases. Recently, studies are being conducted to use EM dampers for vibration control of civil engineering structures.

The ideal model of the EM damper force can be expressed by the damper coefficient and the velocity of the damper. For the ideal damping force and damping coefficient, the time delay and mechanical friction of the damper are not considered. The damping force of the EM damper is proportional to the speed of the structure, like in the conventional mechanical damper. The electromagnetic damping force $F_{d}$ is proportional to the EM damper coefficient $c_{d}$ and applied velocity v:

(1) $$F_{d}=c_{d}\cdot v.$$

**Figure 1:**
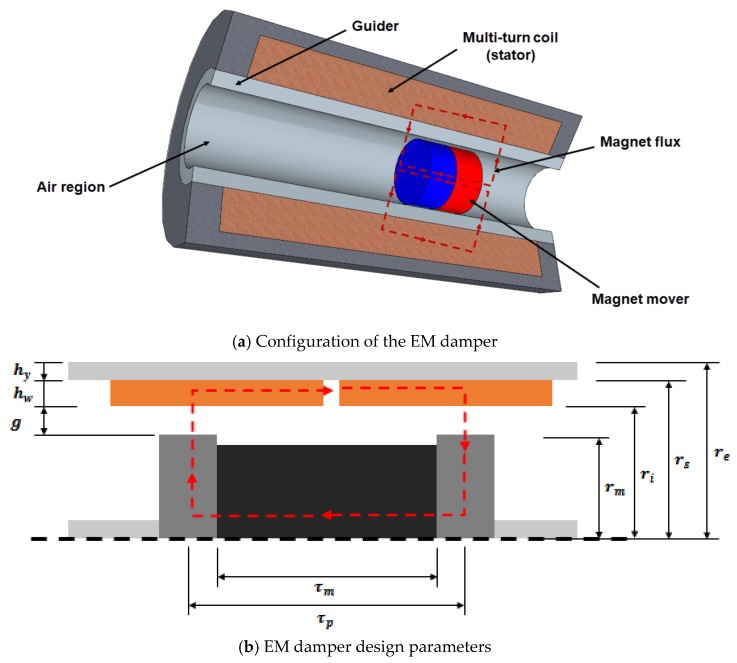
Electromagnetic (EM) damper configuration and design parameters [12].

The damping coefficient of the damper must include a time delay due to the inductance of the damper coil. The damping force-velocity relation including the time delay component of the damper can be expressed as follows:

(2) $$L_{coil}\frac{dF_{d}}{dt}+R_{coil}\cdot F_{d}\;=\;K_{t}^{2}v$$

(3) $$K_{t}\;=\;\frac{\pi pN_{a}r_{m}^{2}B_{rem}\tau_{m}}{\tau_{m}\tau_{f}+r_{m}^{2}\frac{\mu_{rec}}{\mu_{0}}\left(ln\left(\frac{r_{s}}{r_{m}}\right)+\frac{1}{2\mu_{Fe}}+\frac{\tau_{p}\tau_{f}}{\mu_{Fe}h_{y}\left(r_{s}+r_{e}\right)}\right)}$$

where $L_{coil}$ is the inductance of the damper coil, $R_{coil}$ is the resistance of the damper coil, and $K_{t}$ is a motor constant of the damper. Table 1 and Figure 1 show the design parameters of the linear-type EM damper. The parameters in the table include the motor constant of the damper. The detailed derivation of the motor constant $K_{t}$ is given in Palomera-Arias (2005).

Using Equation (2), the damper coefficient, including the time delay, is as follows:

(4) $$c_{d}\;=\;\frac{K_{t}^{2}}{\sqrt{{\left(R_{coil}\right)}^{2}+{\left(\omega L_{coil}\right)}^{2}}}$$

where ω is a frequency of the input excitation. The inductance is influenced by the excitation frequency of the external load. The influence of the inductance is considerable when the external load has a high-frequency band. However, considering that the natural frequencies of most civil infrastructures are in a low-frequency band, the influence of the inductance on the damping coefficient of the EM damper is negligible.

The damping coefficient of the EM damper is influenced by the motor constant, system resistance, and inductance. The maximum damping coefficient of the EM damper, when neglecting the influence of inductance at low frequencies, is as follows:

(5) $$c_{d,max}\;=\;\frac{K_{t}^{2}}{R_{total}}.$$

The resistance $R_{total}$ of the EM damper is the sum of the resistance of the solenoid coil and the resistance of the additional circuitry attached to the outside of the EM damper. Considering $c_{d,max}$, only, the resistance change of the solenoid coil according to the variation of the coil wire thickness is obtained without including the external resistance. The number of turns, wire length, and coil resistance, which are the main properties of a coil, can be estimated by the following equations [17,18]:

(6) $$V_{coil}\;=\;\pi \left(a_{2}{}^{2}-a_{1}{}^{2}\right)h$$

(7) $$\mathrm{FF}\;=\;\frac{V_{w}}{V_{coil}}\;=\;\frac{\frac{\pi }{4}d_{w}{}^{2}l_{w}}{\pi \left(a_{2}{}^{2}-a_{1}{}^{2}\right)h}$$

(8) $$L_{w}\;=\;\frac{4\cdot FF\cdot \left(a_{2}-a_{1}\right)h}{\pi \cdot d_{w}{}^{2}}$$

(9) $$R_{coil}\;=\;\rho \frac{4l_{w}}{\pi \cdot d_{w}{}^{2}}$$

where $V_{coil}$ is the volume of the coil, $V_{w}$ is the volume of the coil wire, $d_{w}$ is the diameter of the coil wire, $l_{w}$ is the total length of the coil wire, $R_{coil}$ is the resistance of the coil, and ρ is a conductivity of the material of the coil.

**Figure 2:**
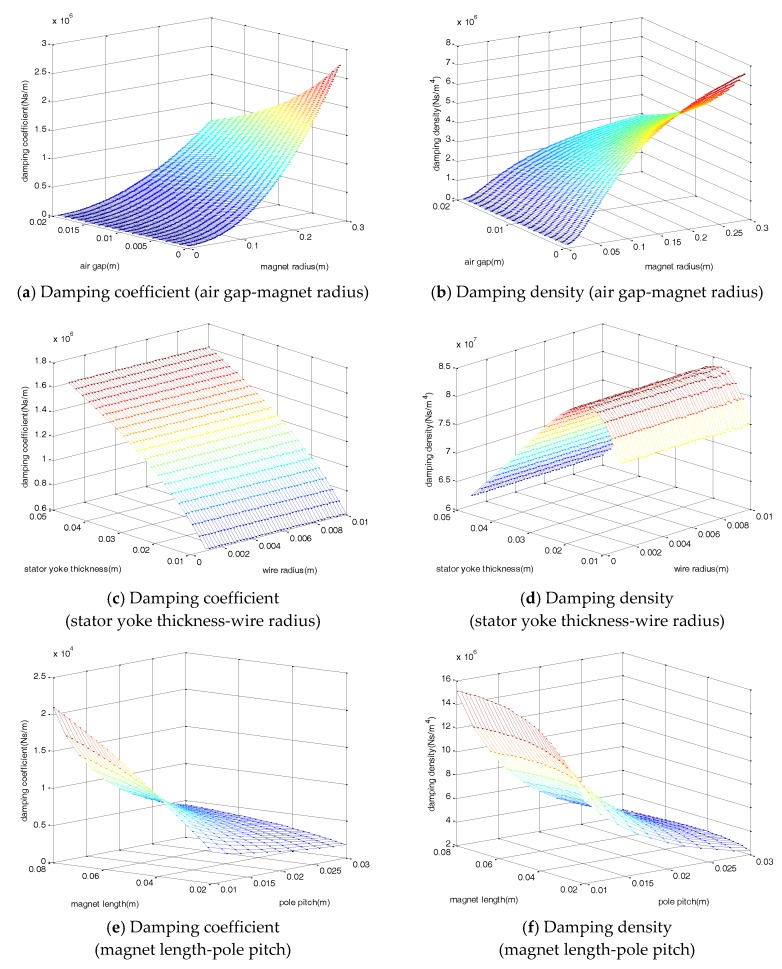
Damping coefficient and density variation.

Figure 2 shows the damping coefficient and density variation of the EM damper. The damping density is obtained by considering both the damping coefficient and volume of the damper. The damping density is the value of the damping coefficient divided by the total volume of the damper, which represents the efficiency according to the total volume of the damper. The damping coefficient and density variation of the magnet radius, air gap, wire radius, stator yoke thickness, magnet length, and pole pitch, were analyzed, all of which have a major influence on the damping coefficient and damping density of the damper. The observed damping coefficient showed a tendency to increase as the air gap and pole pitch decreased, and as magnet radius, magnet length, and stator yoke thickness increased.

Unlike the damping coefficient that continuously increased, the damping density tended to converge to a constant value. For the magnet length and radius, the damping density tended not to increase significantly when a certain value was reached, and the maximum damping density was ~0.02 m for the stator yoke thickness. In the case of the coil, the increase of the radius caused a decrease in the resistance per unit length, but a decrease in the total number of turns and the total length. Conversely, if the radius decreased, the magnitude of the resistance per unit length increased, but the total number of turns and length both increased. By considering all such trends, the coil radius change did not significantly affect the damping coefficient and density. Based on the variation of the damping density according to the design variables, the design parameters shown in Table 2 were selected and the damper was fabricated.

## 3. Characteristic Test of the EM Damper

**Figure 3:**
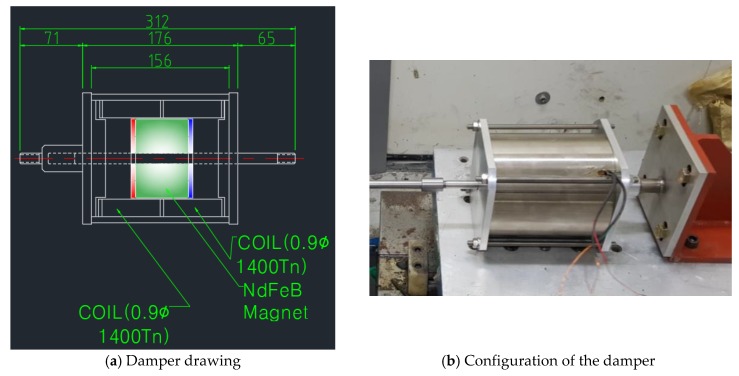
EM damper design drawing and configuration.

The characteristic test of the damper was carried out on the change of the damping force according to the excitation frequency and displacement change. The excitation frequency was changed from 1 to 5 Hz in 1 Hz increments, and the displacement was changed from 6 to 15 mm in 3 mm increments. Table 3 and Figure 3 show the characteristic test cases and damper design with its configuration, respectively. Each column in Table 3 represents the amplitude change of the external load at the fixed frequency. The experimental cases shown in the table mean the amplitude change for the constant frequency of the external load.

**Figure 4:**
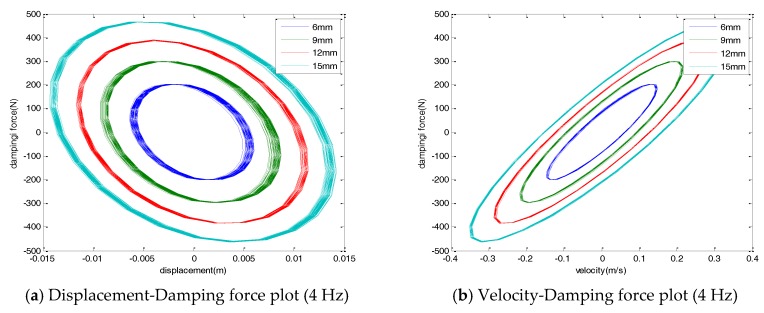
Hysteresis curve (4 Hz, various amplitude).

Figure 4 shows the damping force displacement and damping force velocity with varying excitation amplitudes. As shown in the figures, the damping force of the damper was linearly proportional to the amplitude of the applied load for a constant frequency input. The hysteresis curves of the damper showed an elliptical shape. The direction of the major axis of the ellipse was kept constant and the width of the ellipse was increased when an external load of various amplitudes was applied with a constant frequency.

The force-displacement curve showed a negative stiffness, induced by the magnetic force of the damper. A permanent magnet, which was placed inside a coil, induced an induction current in the coil, producing the Lorentz force. The Lorentz force generated in the damper acted as a damping force of the damper. The Lorentz force acted in the opposite direction to the permanent magnet. Therefore, the force-displacement hysteresis curve of the EM damper showed the negative stiffness.

**Figure 5:**
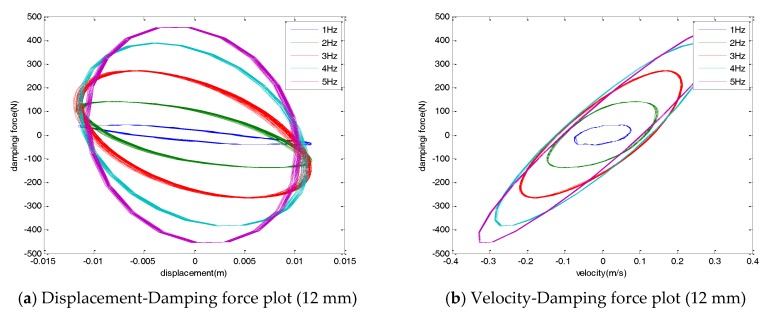
Hysteresis curve (12 mm, varying frequency).

The variation of the damping force as a function of the frequency of the EM damper is shown in Figure 5. For the variations in the external frequency with constant amplitude, the damping force of the damper increased nonlinearly. The major axis of the ellipse of the hysteresis curve shifted when the excitation frequency changed for a constant amplitude. In the displacement-damper force graph, the lengths of the minor and major axes increased simultaneously. That is, as the frequency of the applied load increased, the hysteresis curve gradually became circular. As the excitation frequency increased, the slope of the hysteresis curve increased as the damping coefficient of the damper increased. In addition, as the excitation frequency increased, the area of the hysteresis curve of the displacement-damping force increased. This indicated that the amount of total energy that the damper can dissipate increased.

**Figure 6:**
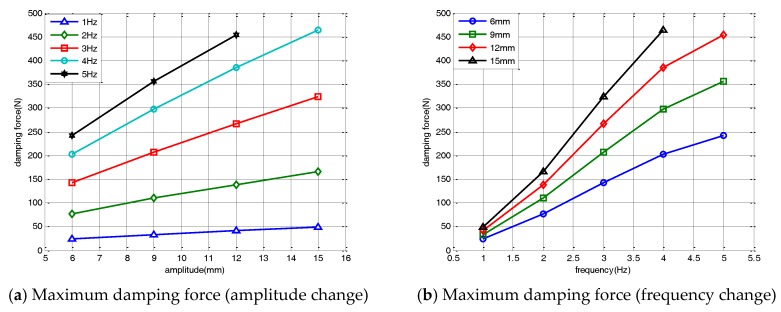
Maximum damping force change.

Figure 6a shows the maximum damping force of the damper for changes in the amplitude of the external load. The maximum damping force of the damper linearly increased as the displacement of the input load increased. The damper force increased linearly as the amplitude increased, even when the input load frequency was changed. Figure 6b shows the variations in the maximum damping force and its nonlinear changes for a varied excitation frequency. In the velocity-force hysteresis curve, the damping coefficient increased as the excitation frequency increased. The nonlinear changes of the hysteresis curve occurred at the input frequency change. For a constant input amplitude, the maximum damping force of the damper increased nonlinearly as the excitation frequency increased. As the magnitude of the change in the magnet flux acting on the coil increased, the damping force of the damper nonlinearly increased as the excitation frequency increased. When the excitation frequency exceeded 5 Hz, the change in the magnet flux reached saturation. The maximum damping force of the damper converged after 5 Hz.

## 4. Hybrid Simulation of the EM Damper

The validation of the vibration-control and energy-harvesting performance of the EM damper was performed using a hybrid simulation. The hybrid simulation is a combination of the experimental method of the physical experiment and a numerical simulation. By combining the numerical simulation and experiment, hybrid simulations can be used to verify full-scale structures or virtual structures that are difficult to verify experimentally [19,20,21]. In this study, a hybrid simulation was constructed using a numerical cable structure and a real damper model. As mentioned previously, the numerical model of the EM damper did not reflect the characteristics of a real EM damper. In this study, the hybrid simulation was constructed to experimentally reflect the behavior of a real EM damper.

In the process of the hybrid simulation, the measurement of the physical experiment and the calculation of the numerical simulation are conducted simultaneously. For the external loads, the response of the numerical cable structure is calculated in the numerical simulation. At the same time, the cable responses at the EM damper position are calculated and applied to the EM damper. Then, the damping force of the EM damper is measured and input to the numerical simulation simultaneously. All processes of calculation and measurement are performed in real-time.

**Figure 7:**
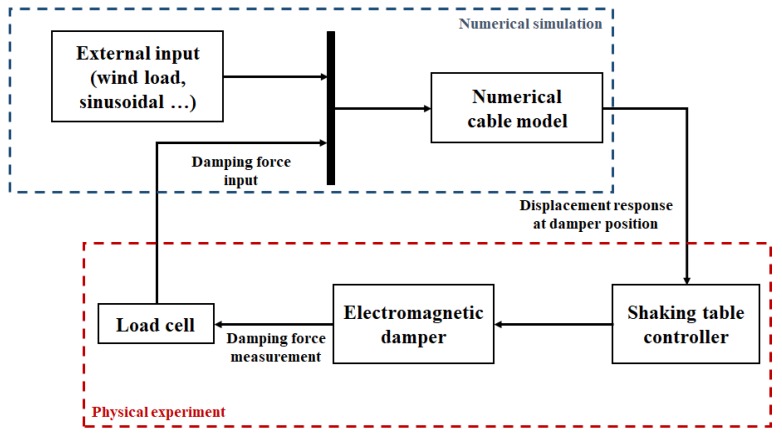
Block diagram of the hybrid simulation.

Since the hybrid simulation had few constraints on the target structure, it is possible to evaluate the performance of the proposed damper by applying the damper to various structures. The physical experiment was performed using a shaking table and the EM damper. The numerical model of the cable was built on the computer. Using this hybrid simulation setup, the response of the cable structure to various external loads, the vibration-control performance of the damper, and the energy-harvesting performance were evaluated. Figure 7 shows a block diagram of the hybrid simulation.

The hybrid simulation was constructed using Matlab Simulink Desktop Real-Time. In the hybrid simulation process, the numerical analysis was conducted by iterating the response of the cable at the damper location and the corresponding damper force. If the computing speed of the simulation could not reach the simulation time, a time delay occurred. However, the time delay was prevented by setting missed ticks. The hybrid simulation was stopped immediately when the number of delayed time steps exceeded the number of missed ticks. In the hybrid simulation, the number of missed ticks was ~100, which is equivalent to 0.1 s. The maximum time delay that could occur in the hybrid simulation was ~0.1 s. Therefore, the time delay due to the computing process of the hybrid simulation was negligible.

**Figure 8:**
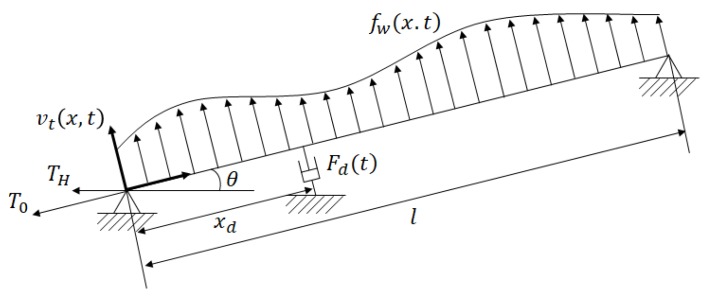
Cable and damper system.

In order to construct the hybrid simulation, a numerical cable model is required. Figure 8 shows the cable structure and the loads acting on the cable. A deformation of the cable occurred, not only due to the external loads but also due to the sag from self-weight. For the cable with a total length of *l,* the deflection due to self-weight at an arbitrary position *x* is given by

(10) $$v_{0}\left(x\right)\;=\;-4f\left(1-\frac{x}{l}\right)\frac{x}{l}\;f\;=\;\frac{mgl^{2}\cos\;\theta }{8T_{0}}$$

where *m* is the mass per unit length, *g* is the acceleration of gravity, *l* is the cable length, and $T_{0}$ is the tension force acting on the cable.

When an external force is applied to the cable, a dynamic displacement occurs, causing additional tension in the cable. If the additional tension caused by the dynamic displacement is T, the equation of motion of the cable is given by

(11) $$m\cdot {\ddot{v}}_{t}\left(x,t\right)+c\cdot {\dot{v}}_{t}\left(x,t\right)-T_{0}\frac{d^{2}}{dx^{2}}v\left(x,t\right)-T\frac{d^{2}}{dx^{2}}v_{0}\left(x\right)=f_{w}\left(x,t\right).$$

The occurrence of instantaneous deformation in a cable is due to the external force and dynamic displacement. This deformation causes additional tension inside the cable. The equation of motion, which takes into account both the dynamic displacement of the cable and the additional strain due to deformation, is expressed by

(12) $$m\cdot {\ddot{v}}_{t}\left(x,t\right)+c\cdot {\dot{v}}_{t}\left(x,t\right)-T_{0}\frac{d^{2}}{dx^{2}}v\left(x,t\right)+\frac{\lambda^{2}}{l^{3}}T_{0}\int_{0}^{l}v\left(x,t\right)dt=f_{w}\left(x,t\right)\;\lambda^{2}={\left(\frac{mgl\cos\;\theta }{T_{0}}\right)}^{2}\frac{EAl}{T_{0}L_{e}}={\left(\frac{8f}{l}\right)}^{2}\frac{EAl}{T_{0}L_{e}}\;L_{e}=\left[1+8{\left(\frac{f}{l}\right)}^{2}\right]l$$

where *E* is the young’s modulus of the cable and *A* is the area of the cable. The transverse deflection of the cable can be approximated by the sum of the sine-shaped assumed shape functions:

(13) $$v\left(x,t\right)\;=\;\overset{n}{\underset{i=1}{\sum }}q_{i}\left(t\right)\phi_{i}\left(t\right)$$

where *n* is the number of modes considered, $q_{i}\left(t\right)$ are the generalized displacements, and $\phi_{i}\left(t\right)$ is a set of the shape functions. According to Johnson et al. (2000), introducing shape functions based on the deflection due to a static force at the damper can reduce the number of terms required for comparable accuracy. Conversely, hundreds of terms in the sine series are generally used as shape functions if this static deflection is not considered, resulting in considerable computation effort [22,23]. The shape function, including the deflection due to a static force, can be expressed by

(14) $$\phi_{1}\left(x\right)\;=\;\{\begin{array}{c} \frac{x}{x_{d}}\;\left(0\leq x\leq x_{d}\right)\\ \frac{l-x}{l-x_{d}}\;\left(x_{d}\leq x\leq l\right) \end{array}\;\phi_{i}\left(x\right)=\sin\left(\pi i\frac{x}{l}\right)\;\left(i=2,3,\cdots ,n\right)$$

Using Equations (12) and (14), a non-dimensional equation of motion of the cable and its matrix form can be expressed as [24]:

(15) $$m\int_{0}^{l}\phi_{i}\phi_{j}dx\cdot \ddot{q_{j}}+c\int_{0}^{l}\phi_{i}\phi_{j}dx\cdot \dot{q_{j}}+T_{0}\int_{0}^{l}{\phi_{i}}^{\prime }{\phi_{j}}^{\prime }dx\cdot q_{j}+\frac{\lambda^{2}}{l^{3}}T_{0}\int_{0}^{l}\phi_{i}dx\int_{0}^{l}\phi_{j}dx\cdot q_{j}\;\;=\int_{0}^{l}\phi_{i}f_{w}\left(x,t\right)dx$$

(16) $$M\ddot{q}+C\dot{q}+Kq=F+F_{d}$$

where $M=\left[m_{ij}\right]=\left[m\int_{0}^{l}\phi_{i}\phi_{j}dx\right]$ is the mass matrix, C=αM+βK is the damping matrix calculated form Rayleigh damping, and $K=\left[k_{ij}\right]=\left[T_{0}\int_{0}^{l}{\phi_{i}}^{\prime }{\phi_{j}}^{\prime }dx+\frac{\lambda^{2}}{l^{3}}T_{0}\int_{0}^{l}\phi_{i}dx\int_{0}^{l}\phi_{j}dx\right]$ is the stiffness matrix. The properties of the cable damper model are shown in Table 4.

**Figure 9:**
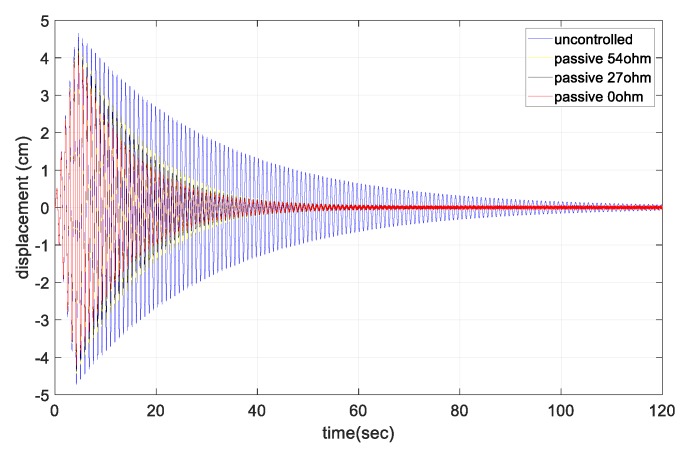
Free-vibration response of the cable (middle point, ½ L).

The free-vibration response at the center point of the cable according to the change in the external resistance is shown in Figure 9. As the larger external resistance was connected, the smaller control force of the damper was shown. In the free-vibration test, the vibration dissipation capacity of the damper sharply decreased as the external resistance increased. In the uncontrolled case without the damper, the cable damping ratio was 0.48%. For the short circuit case with zero external resistance, the damping ratio was increased by ~3 to 1.32%. As the external resistance increased, the damping ratio gradually decreased to ~1%. Table 5 shows the damping ratio change of the cable due to the variations in the external resistance.

**Figure 10:**
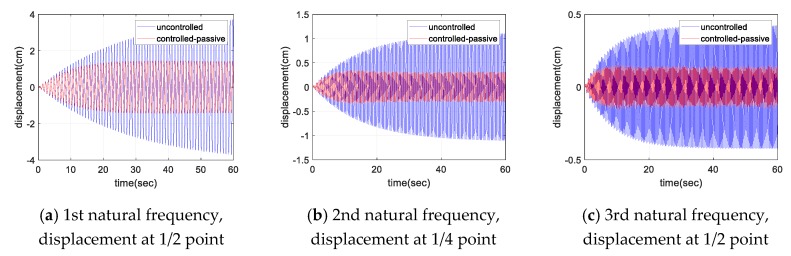
Displacement response of the cable for sinusoidal input.

**Figure 11:**
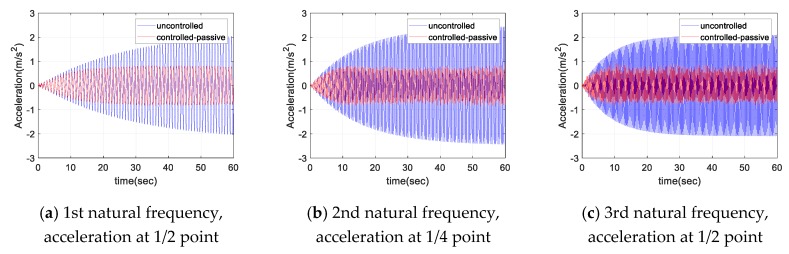
Acceleration response of the cable for sinusoidal input.

**Figure 12:**
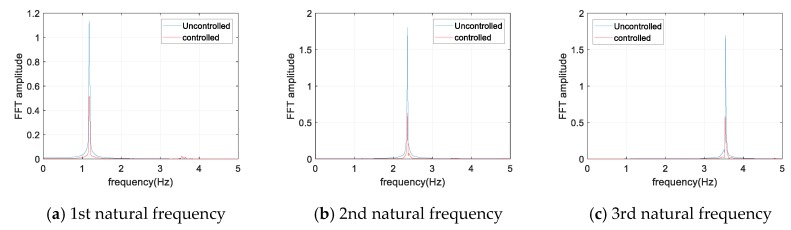
FFT plot for sinusoidal input.

Figure 10 and Figure 11 show the displacement and acceleration response of the cable with the natural frequency excitation, respectively. As shown in the figures, for a constant sinusoidal input load, the damper showed a certain level of damping performance. For each sinusoidal input load, the magnitude of the maximum response of cable was reduced to less than half in both the displacement and acceleration responses. Figure 12 shows the frequency domain response for each sinusoidal input. The magnitude of the response was reduced to less than half, even in the frequency domain with respect to each natural frequency. At least 50% of the response attenuation was shown for all the frequency input. The results of the sinusoidal input are shown in Table 6. The uncontrolled, controlled, and decay ratio of displacement, acceleration, and FFT amplitude are shown in Table 6.

**Figure 13:**
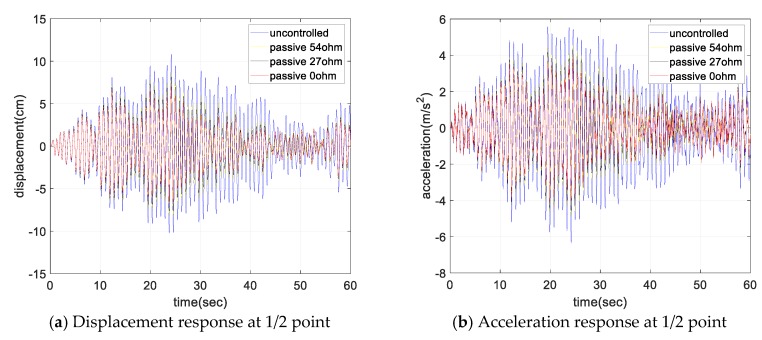
5 m/s mean wind speed, 1/2 L response.

**Figure 14:**
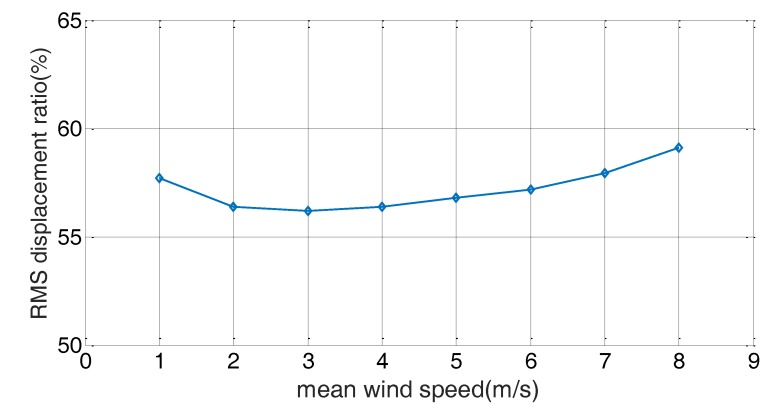
RMS response ratio for mean wind speed variation.

Figure 13 shows the displacement and acceleration response of the cable as the resistance changed. Similar to the free-vibration test, the damper showed better damping performance as the external resistance decreased. For the short circuit case, the response decreased by more than 40% for all values of mean wind speed. Figure 14 shows the change in RMS value of the cable displacement for the variations in the mean wind speed. It represents the ratio of the RMS response for the uncontrolled case without damper. The RMS response was reduced by more than 40% for all values of mean wind speed, and the RMS response reduction was a maximum at a mean wind speed of 3 m/s.

**Figure 15:**
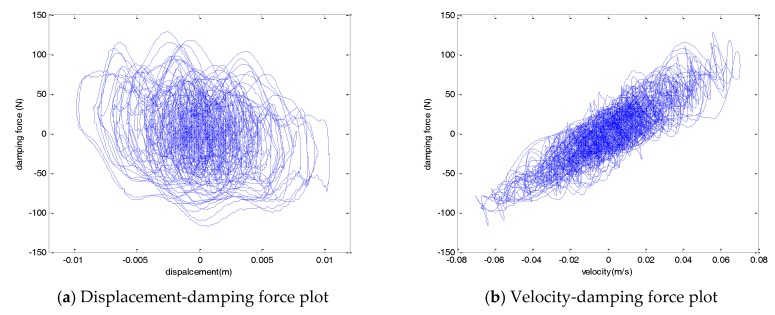
Hysteresis curve (mean wind speed of 5 m/s).

Figure 15 shows the hysteresis curve for a mean wind speed of 5 m/s. Unlike the characteristic test, the wind load acting on the cable contained various frequency components. As the cable in which the wind load was applied was excited by various frequency components, the hysteresis curve of the damper also showed characteristics including various frequency components. Both the displacement-force and velocity-force graphs did not exhibit a constant ellipse trajectory like the sinusoidal input. As the various frequency components were contained, several ellipsoidal trajectories are shown in the hysteresis curve.

**Figure 16:**
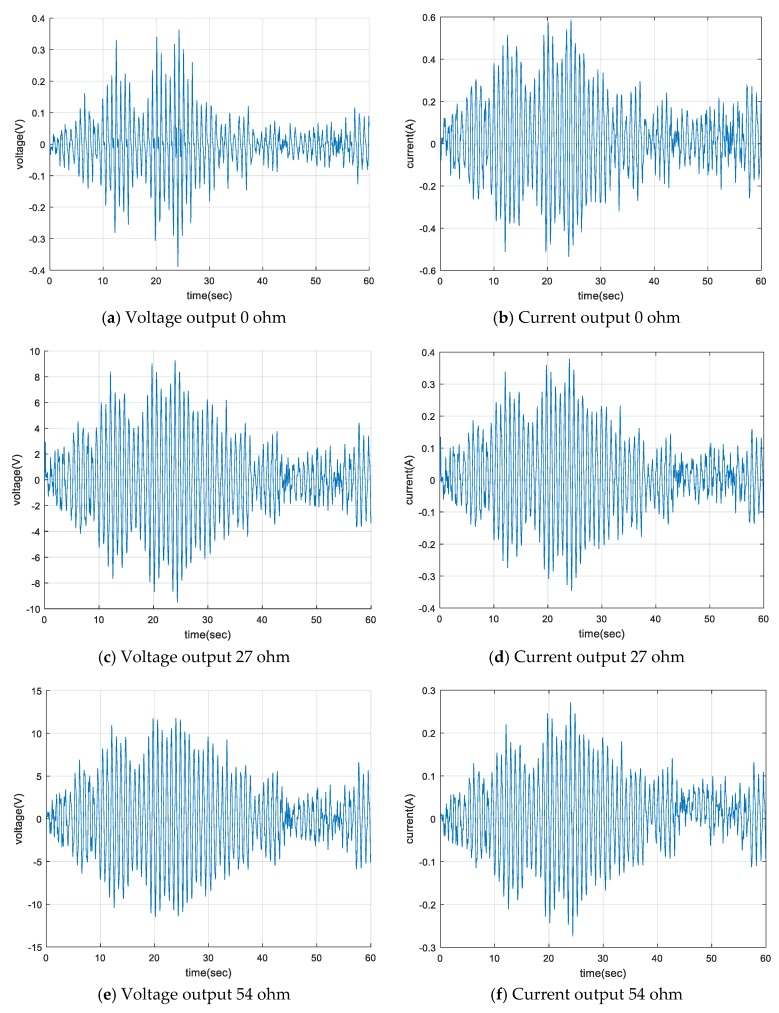
Voltage and current output.

The voltage and current output of the EM damper according to the external resistance change for a 5 m/s mean wind speed are shown in Figure 16 and Table 7. For the case of zero external resistance, the output current from the damper was a maximum. However, the power output from the damper was a minimum because of the relatively small voltage output. As the magnitude of the external resistor increased, the magnitude of the current generated in the damper gradually decreased. However, the voltage generated by the damper was relatively increased. The magnitude of the power generated by the damper gradually increased as the magnitude of the external resistance increased.

The magnitude of the power generated by the EM damper was related to the damper speed and the magnitude of the external load. The damping coefficient of the damper decreased as the external resistance increased. For a constant magnitude of the external loads, the larger the external resistance applied to the damper, the greater the magnitude of the relative velocity of the permanent magnet and solenoid coil of the damper. As a result, the magnitude of the voltage that could be obtained from the damper increased, but the magnitude of the current gradually decreased. The magnitude of the total power gradually increased, because the magnitude of the voltage rise was relatively larger than the amount of the current reduction, and it finally converged to a specific value.

**Figure 17:**
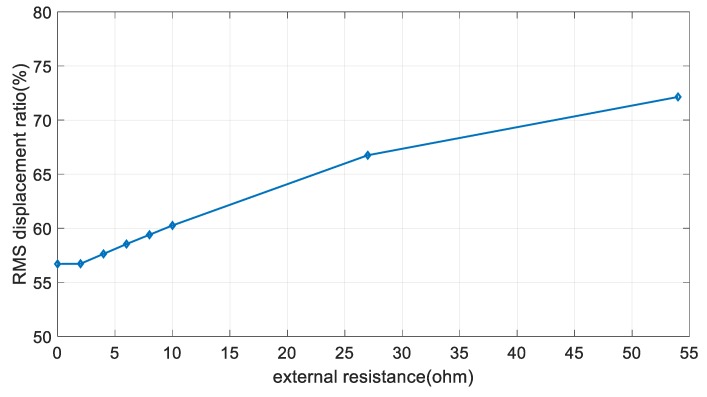
RMS response ratio for various external resistance (mean wind speed of 5 m/s).

According to the Korea Meteorological Administration (KMA), the mean wind speed in the past 5 years from 2010 was ~5.3 m/s at the 2nd Jindo bridge. The proposed EM damper can acquire power of more than 100 mW when using an external resistance of 4 ohm or more, at a mean wind speed of 5 m/s. According to the Park et al. (2010), ~42 mW of power is required when two measurements are performed on the Imote2 wireless sensor [25]. The proposed system shows a sufficient energy-harvesting performance to drive the wireless monitoring sensor, even considering the power loss of the energy-harvesting circuit. However, as shown in Figure 17, if the size of the external resistance is increased to improve the energy-harvesting performance, the vibration-control performance of the cable is remarkably decreased. There is a limit to simultaneously satisfy the energy-harvesting and vibration-control performances. Therefore, it is necessary to develop a proper system operation plan according to changes in the external loads. The proposed EM damper is operated as an energy-harvesting device for the ambient wind loads. Considering the mean wind speed acting on the bridge, a sufficient power can be acquired to drive the monitoring sensors. In addition, the battery cell can be used for storing the remaining power, which can be used when the operation of the energy-harvesting system is difficult. The EM damper is used as the vibration-control system for external loads that cause excessive vibration of cables, such as typhoons and earthquakes. If excessive vibration occurs in the cable, the system is operated in the direction of maximizing the vibration-control performance of the EM damper by minimizing the energy-harvesting performance.

## 5. Conclusions

In this study, the EM damper was first fabricated as a vibration-control method of a cable structure, and characteristic and performance tests were conducted. Contrary to previous studies, the performance of the EM damper was analyzed using a hybrid simulation incorporating the bridge cable properties to reflect the actual characteristics of the cable structure. Also, the energy-harvesting performance was analyzed by using the mean wind speed acting on the actual bridge. In order to evaluate the feasibility of the damper, the performance of the EM damper was evaluated by reflecting actual wind load.

In order to evaluate the applicability of the EM damper to the cable structure, a characteristic test of the damper and a performance evaluation using a hybrid simulation were carried out. As a result of the characteristic test, a linear damping force change was confirmed in the amplitude change of the external load, and nonlinear damping force change was observed in the frequency variation.

The hybrid simulation results showed that the EM damper had a certain level of damping performance for the cable structure. In the free-vibration test, the damper showed a maximum damping ratio of 0.0132 at zero external resistance. As the external resistance increased, the damping ratio gradually decreased. It was confirmed that the attenuation performance was more than 50% in both the frequency and time domains for the sinusoidal input of the first to third natural frequencies. The RMS displacement reduction of the cable was 42–44% in the case of wind load. As the magnitude of the external resistance increased, the cable vibration-control performance decreased, and the magnitude of the power generated by the damper gradually increased.

The applicability of the EM damper to a cable structure needs to be approached, not only from the vibration-control performance, but also from the maintenance aspect using the generated power. The vibration-control performance of the damper was insufficient compared to conventional damper systems. However, as excessive vibration of a cable can be reduced to an appropriate level, it could be applied to a cable structure in which vibration is continuously generated. In addition, as the damping coefficient of the damper can be adjusted using an external resistance, it could be utilized as a semi-active system, and better vibration-control performance is expected.

The energy-harvesting performance of the damper gradually increased as the external resistance increased. However, as the external resistance increased, the vibration-control performance of the damper decreased; therefore, it is necessary to maintain a proper vibration-control performance level.

According to the KMA, the mean wind speed was ~4.8 m/s at the 2nd Jindo bridge, and 80% of the one-minute wind speed was more than 3 m/s. This can be expected to generate sufficient power to operate a wireless sensor for monitoring. The wind speed above 10 m/s was ~4.6%. Most wind speeds are expected to be used for energy harvesting. As the power consumption of low-power sensors used for civil structure monitoring is several tens of mW, the power obtained from the damper is expected to be used as a power source for monitoring sensors.

## Figures and Tables

**Table 1:** Electromagnetic (EM) damper design parameter [12].

Parameter	Symbol	Description
**Pole pitch**	$\tau_{p}$	Distance between changes in polarity
**Magnet length**	$\tau_{m}$	Actual length of magnet
**Pole shoe width**	$\tau_{f}$	Width of the pole shoes
**Air gap**	g	Distance between the mover and armature windings
**Number of poles**	p	Even number of poles in the machine
**Coil width**	$\tau_{w}$	Width of each coil in the armature
**Wire radius**	$r_{w}$	Radius of the coil wire
**Coil turns**	$N_{w}$	Number of turns on each coil
**Active coil turns**	$N_{a}$	Number of turns on each coil intercepted by the pole shoe flux
**Mover radius**	$r_{m}$	Radius of the outside surface of the magnets
**Armature radius**	$r_{i}$	Radius of the inside surface of the armature
**Stator yoke radius**	$r_{s}$	Radius of the inside surface of the stator yoke
**Machine radius**	$r_{e}$	Radius of the outer surface of the motor
**Yoke thickness**	$h_{y}$	The thickness of the armature shell

**Table 2:** Design parameters of the EM damper.

Parameter	Symbol	Dimension
**Pole pitch**	$\tau_{p}$	0.07 m
**Magnet length**	$\tau_{m}$	0.06 m
**Air gap**	g	0.001 m
**Number of poles**	p	1
**Coil height**	$h_{w}$	0.02 m
**Wire radius**	$r_{w}$	0.0045 m
**Coil turns**	$N_{w}$	1400 turn
**Coil resistance**	R	27 Ω
**Yoke thickness**	$h_{y}$	0.02 m

**Table 3:** Characteristic test cases.

	Amplitude	Frequency	External Resistance
Case 1–4	6 mm, 9 mm, 12 mm, 15 mm	1 Hz	0 ohm
Case 5–8	6 mm, 9 mm, 12 mm, 15 mm	2 Hz	0 ohm
Case 9–12	6 mm, 9 mm, 12 mm, 15 mm	3 Hz	0 ohm
Case 13–16	6 mm, 9 mm, 12 mm, 15 mm	4 Hz	0 ohm
Case 17–19	6 mm, 9 mm, 12 mm	5 Hz	0 ohm

**Table 4:** Properties of the cable model.

Properties	Value
Mass per unit length	22.1 kg/m
Cable length	20 m
Diameter	54.6 mm
Cable tension	50 kN
Young’s modulus	189 MPa
Inclination	8.38°
Cross section area	0.0023${\;m}^{2}$
Location of damper	5% length of cable

**Table 5:** Damping ratio variation.

Case	Damping Ratio
Uncontrolled	0.0048
Controlled 0 ohm	0.0132
Controlled 27 ohm	0.0113
Controlled 54 ohm	0.0100

**Table 6:** Response decay ratio for sinusoidal input (decay ratio).

	Displacement (cm)	Acceleration ($m/s^{2}$)	FFT Amplitude
1st natural frequency	3.717	2.044	1.136
	1.447 (61.07%)	0.839 (58.95%)	0.520 (54.26%)
2nd natural frequency	1.106	2.434	1.800
	0.343 (68.99%)	0.856 (64.83%)	0.629 (65.05%)
3rd natural frequency	0.424	2.092	1.670
	0.152 (64.15%)	0.934 (55.35%)	0.572 (65.75%)

**Table 7:** RMS power output.

	RMS Voltage (V)	RMS Current (A)	RMS Power (mW)
0 ohm	0.0801	0.1881	15.1
2 ohm	0.3679	0.1841	67.7
4 ohm	0.6865	0.1730	118.8
6 ohm	0.9799	0.1647	161.3
8 ohm	1.2486	0.1571	196.1
10 ohm	1.4984	0.1506	225.7
27 ohm	2.9981	0.1170	350.8
54 ohm	4.3179	0.0863	372.6
